# Social Media and Microbiology Education

**DOI:** 10.1371/journal.ppat.1001095

**Published:** 2010-10-21

**Authors:** Vincent R. Racaniello

**Affiliations:** Department of Microbiology & Immunology, Columbia University Medical Center, New York, New York, United States of America; The Fox Chase Cancer Center, United States of America

Social media consists of Internet technologies that allow users to create and share content, and to foster dialogues among other users. Examples include software applications for communication (blogging, social networking, discussion forums), collaboration (wikis, social bookmarking), and multimedia (sharing photographs, video, and livecasting). In the world of science, social media is becoming an increasingly integral component of both research and education. My experience with two types of social media, blogging and podcasting, has convinced me that scientists must embrace these applications to enhance research, and to better communicate their work to the public.

## An Unconventional Textbook

My foray into social media began 10 years ago when I decided to co-author a virology textbook [1]. This book is unusual because it does not teach virology virus by virus, but according to the steps of the infection cycle. While conventional virology textbooks contain chapters on individual viruses, *Principles of Virology* focuses on the processes of infection, such as virus entry into cells, pathogenesis, and evolution. Because each author wrote individual chapters, it was necessary for each of us to learn about many different viruses, not just those that are the subjects of our research. This writing experience provided each author with a broad view of, as well as an enduring interest in, the principles, processes, and strategies of virology.

## Virology Blog

Although a broad understanding of virology was very useful for teaching, I only engaged ten to 20 students each year. I began to look for ways to reach a broader audience, and in 2004 began virology blog (http://www.virology.ws/). A blog (portmanteau for Web log) is a Web site containing entries in a journal style displayed in a reverse chronological order. I chose the blog format because it is designed to facilitate rapid online publication with a minimum of technical expertise. The ease and zero cost of blogging have enticed millions of individuals to become “bloggers”. While the name has become synonymous with a critical and incendiary non-professional, the form has become highly refined and embraced by many skilled writers.

My first post at virology blog, “Are viruses living?”, went unread for months. But I kept writing, imagining that I was teaching the world about viruses. Eventually someone wrote a comment on that first post. I kept writing, more comments appeared, and the audience grew steadily. Today there is an average of 1,500 visitors to the site each weekday, with peaks of 10,000 visitors on certain days, such as when the 2009 H1N1 influenza vaccine was released. In the past year virology blog has had nearly 500,000 readers from 214 countries, which means that I am reaching more individuals than I have taught in my entire teaching career. Those statistics are surprising for a highly technical and specialized field. Simplicity is a key to engaging a broad readership. Virology blog posts are no longer than 500–1,000 words, are accompanied by an illustration, and are free of jargon or complex abbreviations.

One goal of virology blog is to explain news stories about viruses. When a rotavirus vaccine was withdrawn in the United States because it contained a viral contaminant, I wrote an article explaining the findings and what they meant. The paper describing the findings had not yet been published, but the author sent me the manuscript after reading the blog article, and my readers benefited from access to “inside information”. I also explain the methods of virology, such as neutralization assays and virus titration. And, from time to time it is useful to speculate: what will influenza virus H1N1 do next? Does XMRV cause chronic fatigue syndrome?

Some visitors reach virology blog by typing the URL into a browser; but most arrive after a Google search. Some popular search terms leading to virology blog include HeLa cells, influenza H1N1, plaque assay, virology blog, and virology. Each article is also labeled with key words to facilitate discovery by search engines. Google Images is another important referral source, since each post has at least one clearly labeled illustration. Many users arrive from Facebook—with 300,000,000 users, it is a significant starting point for visitors seeking science information.

## What's Social about a Blog?

On virology blog, readers can also be content creators. The first article, “Are viruses living?”, now has 60 different comments. One reader wrote, “For me they are living, I think you're being unnecessarily restrictive”. Others soon weighed in with questions that were frequently answered by other readers. “And what about prions?” wrote another. In the discussion section, readers have a dialogue among themselves, inspired by the topic of the post. A significant advantage of the blog format is that all readers can have a conversation, and learn, free of charge, from an expert in a particular field.

## Open Science

After being a principal investigator for 28 years, I still enjoy working in the laboratory. I decided to share on virology blog the results of experiments I initiated to identify the target in the rhinovirus genome of zinc inhibition [2]. Such disclosure would be an effective way to teach the public how scientific experimentation proceeds, and especially convey the high failure rate. To my surprise, readers weighed in with suggestions for experiments, many of which I implemented. By making research results public, crowd sourcing can be used to accelerate science progress.

Although many individuals contribute comments to virology blog, there are many who read but do not participate. A well-known Internet statistic indicates that 90% of a site's readers lurk, 9% contribute a little, and just 1% contribute regularly [3]. But it is not difficult to engage those silent participants—you just have to ask. Sometimes I'll publish a pop quiz—“Can you name one scientist?”—and hundreds will respond. Another good way to engage readers is to take a poll. So far there are 717 responses to the question “Are viruses living?” You can find the results at http://www.virology.ws/are-viruses-alive/.

## This Week in Virology

Conversations are an essential part of the scientific process. We all have them—at lunch, during seminars, in the hallways. Some of them are terrific, but few people get to hear them. That's unfortunate, because chats among scientists can be wonderful learning opportunities. What if they could be recorded and distributed on the Internet? Then it would be called a podcast.

A podcast is audio content distributed on the Internet. The name comes from the fact that the first podcasts were made popular by the Apple iPod, but they can be played online or with any digital audio player. While listening to podcasts during my long commute, I heard Leo Laporte, founder of the podcast network http://twit.tv/, say “Anybody can make a podcast, you just need to be passionate about something.” That described my feelings about virology, so I recruited my colleague Dickson Despommier, a parasitologist, and *This Week in Virology* (TWiV) (http://www.twiv.tv/) was born. We began recording weekly conversations about viruses, put them in the podcast directory in the iTunes music store, and within a few months we had hundreds of listeners. Soon we added two more hosts: Alan Dove, a science writer, and a second virologist, Rich Condit. Guests are also an important part of the podcast: we've had the likes of Peter Palese, Adolfo Garcia-Sastre, Matt Frieman, Lynn Enquist, David Bloom, Grant McFadden, and Eric Delwart.

TWiV is a conversation among scientists made accessible to everyone by the Internet. It is also a free science education. Each episode is accompanied by a blog entry, which includes a title, a brief description, and links to background articles. On a typical TWiV episode, we'll explain the week's stories about viruses, deconstruct complex journal articles, and answer listener questions. For the “weekly science pick”, the hosts each name a science-related book, Web site, movie, or museum that they have enjoyed.

## TWiV Social

We've made TWiV social by answering listener questions. On the first episode, we encouraged listeners to send in their comments and questions, and we read them on each episode. There are so many that every few months we do an episode devoted to reader feedback—a recent one was called “Please Mr. Postman”. There is no better testimony to the effectiveness of TWiV than listener feedback. One listener wrote, “I have run a homebuilding company. I'm not a scientist, but in a Jeffersonian way, I've always had a burning desire to enlighten myself. I want you to know that using jargon is okay. I usually Google terms I don't understand; for me this is far superior to having the podcast dumbed down to a junior high school level.” Another listener wrote, “This is science podcasting at its very best”. Others admit that they have considered entering the field of virology because of TWiV. One commented, “If I were younger, I would become a virologist”. More comments can be found at http://www.twiv.tv/letters/. The measure of an effective teacher is whether he or she can encourage other people to enter the field. By these criteria, TWiV is a success.

Who listens to TWiV? Five to ten thousand people download each episode, including high school, college, and graduate students, medical students, post-docs, professors in many fields, information technology professionals, health care physicians, nurses, emergency medicine technicians, and non-professionals: sanitation workers, painters, and laborers from all over the world.

The success of TWiV inspired us to create a second podcast, *This Week in Parasitism* (TWiP) (http://www.microbeworld.org/twip/). The philosophy is the same as TWiV: to provide a conversation among experts that will teach the public about eukaryotic parasites. The number of TWiP listeners is growing faster than TWiV, proving again that the public is hungry for excellent science education.

## A Science Podcast Network

The success of virology blog, TWiV, and TWiP demonstrates that scientists can successfully engage the public and teach them about complex science. These efforts are only a small part of what is possible with current social media software applications. TWiV and TWiP are only available as audio programs, but we have experimented with video and the results have been encouraging. A video podcast allows inclusion of visual material that might be best suited for explanation of more complex concepts. It would also be of interest to produce “live” versions of our podcasts, broadcast on the Internet during recording. This format would allow listeners to submit questions as the discussion proceeds. Successful interactive science podcasts include *Futures in Biotech* (http://twit.tv/fib/) and *Dr. Kiki's Science Hour* (http://www.twit.tv/kiki/). It should be possible to engage the public in life science education by using a network of live, interactive video podcasts hosted by specialists in a wide range of fields. If such podcasts attract large numbers of viewers they might be sustainable solely by advertising revenue, providing the public with a free science education.

Other scientists have embraced social media to inform the public about their respective fields (see Box 1), but those in the field of microbiology are scarce. Blogs and podcasts are distributed free of charge, but they are not without cost, as they require time to prepare. But the time spent educating the public about science is a priceless investment.

**Box 1. Selected Microbiology Blogs and Podcasts**

**Blogs**

- Mystery Rays from Outer Space—Meddling with things mankind is not meant to understand (http://www.iayork.com/MysteryRays/)
- Research Blogging—Aggregating academic blog posts about peer-reviewed research (http://www.researchblogging.org/)
- Small Things Considered—The microbe blog (http://schaechter.asmblog.org/)
- MicrobiologyBytes—The latest news about microbiology (http://www.microbiologybytes.com/blog/)
- ViroBlogy—Up-to-date virology-related posts (http://rybicki.wordpress.com/)

**Podcasts**

- *Futures in Biotech* (http://www.twit.tv/fib/)
- *Dr. Kiki's Science Hour* (http://www.twit.tv/kiki/) (The preceding podcasts feature interviews with a wide range of scientists, occasionally in the field of microbiology)
- *Persiflager's Infectious Disease Puscast*—A bimonthly review of the infectious disease literature (http://moremark.squarespace.com/puscast-pacid-podcast/)
- *MicrobeWorld Video*—Highlights of the latest in microbiology from the American Society for Microbiology (http://www.microbeworld.org/index.php?option=com_content&view=category&layout=blog&id=36&Itemid=146)

## References

[1] Flint SJ, Enquist LW, Racaniello VR, Skalka AM (2009). Principles of virology..

[2] Korant BD, Kauer JC, Butterworth BE (1974). Zinc ions inhibit replication of rhinoviruses.. Nature.

[3] Nonnecke B, Preece J (2000). Lurker demographics: Counting the silent.. Proceedings of CHI 2000.

